# Racial and Ethnic Disparities in Providing Guideline-Concordant Care After Hip Fracture Surgery

**DOI:** 10.1001/jamanetworkopen.2024.29691

**Published:** 2024-08-27

**Authors:** Mikhail A. Bethell, Kenneth A. Taylor, Colleen A. Burke, Denise E. Smith, Lulla V. Kiwinda, Megan Badejo, Malcolm R. DeBaun, Mark Fleming, Christian A. Péan

**Affiliations:** 1 Duke University School of Medicine, Durham, North Carolina; 2 Duke University School of Medicine, Department of Orthopaedic Surgery, Durham, North Carolina; 3 Duke University School of Medicine, Duke Clinical Research Institute, Durham, North Carolina; 4 Duke University School of Medicine, Department of Population Health Sciences, Durham, North Carolina

## Abstract

**Importance:**

Institutions have adopted protocol-driven standardized hip fracture programs (SHFPs). However, concerns persist regarding bias in adherence to guideline-concordant care leading to disparities in implementing high-quality care for patients recovering from surgery for hip fracture.

**Objective:**

To assess disparities in the implementation of guideline-concordant care for patients after hip fracture surgery in the American College of Surgeons National Surgical Quality Improvement Program (ACS-NSQIP) Targeted Hip Fracture (THF) Database.

**Design, Setting, and Participants:**

This cross-sectional study was conducted using the ACS-NSQIP THF database from 2016 to 2021 for patients aged 65 years and older with hip fractures undergoing surgical fixation. Care outcomes of racial and ethnic minority patients (including American Indian or Alaska Native, Asian, Black or African American, Native Hawaiian or Pacific Islander, or multiple races and Hispanic ethnicity) were compared with non-Hispanic White patients via risk difference, stratified by care institution SHFP status. Modified Poisson regression was used to measure interactions. Statistical analysis was performed from November 2022 to June 2024.

**Main Outcomes and Measures:**

The primary outcomes of interest encompassed weight-bearing as tolerated (WBAT) on postoperative day 1 (POD1), venous thromboembolism (VTE) prophylaxis, bone-protective medication, and the presence of SHFP at the institution.

**Results:**

Among 62 194 patients (mean [SD] age, 82.4 [7.3] years; 43 356 [69.7%] female) who met inclusion criteria and after multiple imputation, 11.2% (95% CI, 10.8%-11.5%) were racial and ethnic minority patients, 3.3% (95% CI, 3.1%-3.4%) were Hispanic patients, and 92.0% (95% CI, 91.7%-92.2%) were White. Receiving care at an institution with an SHFP was associated with improved likelihood of receiving guideline-concordant care for all patients to varying degrees across care outcomes. SHFP was associated with higher probability of being WBAT-POD1 (risk difference for racial and ethnic minority patients, 0.030 [95% CI, 0.004-0.056]; risk difference for non-Hispanic White patients, 0.037 [95% CI, 0.029-0.45]) and being prescribed VTE prophylaxis (risk difference for racial and ethnic minority patients, 0.066 [95% CI, 0.040-0.093]; risk difference for non-Hispanic White patients, 0.080 [95% CI, 0.071-0.089]), but SHFP was associated with the largest improvements in receipt of bone-protective medications (risk difference for racial and ethnic minority patients, 0.149 [95% CI, 0.121-0.178]; risk difference for non-Hispanic White patients, 0.181 [95% CI, 0.173-0.190]). While receiving care at an SHFP was associated with improved probability of receiving guideline-concordant care in both race and ethnicity groups, greater improvements were seen among non-Hispanic White patients compared with racial and ethnic minority patients.

**Conclusions and Relevance:**

Older adults who received care at an institution with an SHFP were more likely to receive guideline-concordant care (bone-protective medication, WBAT-POD1, and VTE prophylaxis), regardless of race and ethnicity. However, the probability of receiving guideline-concordant care at an institution with an SHFP increased more for non-Hispanic White patients than racial and ethnic minority patients.

## Key Points

**Question:**

Does provision of guideline-concordant care following surgery for hip fracture differ by race and ethnicity?

**Findings:**

This cross-sectional study of 62 194 patients found that institutions with standardized hip fracture protocols were associated with higher likelihood of receiving guideline-concordant care for all patients, particularly in weight-bearing as tolerated on the first postoperative day, as well as in the prescription of venous thromboembolism prophylaxis and bone-protective medication. However, the benefit was greater among non-Hispanic White patients compared with racial and ethnic minority patients.

**Meaning:**

This study’s results suggest that disparities exist in guideline-concordant care for racial and ethnic minority patients after hip fracture surgery, emphasizing the need for targeted interventions to improve care equity.

## Teaser

This cross-sectional study investigates racial and ethnic disparities in the implementation of guideline-concordant care after hip fracture surgery among patients aged 65 years and older.

## Introduction

In the United States, racial disparities are present in orthopedics across various subspecialties in the United States.^1,2,3^ A survey of orthopedic surgeons indicated that a mere 9% acknowledged the potential adverse impact of racial and ethnic bias on the quality of orthopedic care.^4^ Nonetheless, Black and Hispanic patients aged 65 years or older experience longer delays to orthopedic surgery, extended hospital stays, increased rates of revision surgical procedures, and higher readmission rates after hip fracture compared with their White counterparts.^5,6,7,8^ Furthermore, Black patients face longer times for radiographic workups and are more likely to receive nonsurgical treatment for fractures despite similar fracture severity and injury burden.^9^ These racial and ethnic disparities add to the already high direct costs of hip fracture in the United States, estimated at the episodic level to be $52 512, resulting in a staggering annual economic burden of $5.96 billion.^10^

Gender also interacts with other social structures of inequality, influencing the health and health care experiences of both women and men. Women routinely receive lower quality care than men across different health care settings.^11,12,13,14,15,16,17^ Among older adults, women are more likely to experience a hip fracture,^18^ however, one study found that women were less likely to receive perioperative geriatric care and anesthesia consultations than men.^19^ Despite differences in care quality between women and men among older adults with hip fractures, men with hip fractures are younger, less healthy, and have a higher incidence of postoperative mortality and morbidity.^20,21^ Social identities based on gender and/or biological sex are not mutually exclusive from those based on race and ethnicity,^22,23^ yet there is a paucity in the literature exploring the intersectionality of these social identities as it relates to hip fracture care quality outcomes.

Improving hip fracture outcomes through comprehensive care models and emphasizing the prevention of secondary fractures can alleviate the economic burden of hip fractures by decreasing undesirable postsurgical outcomes that result in added cost.^24^ Institutions have endeavored to optimize patient care after hip fracture by implementing protocol-driven standardized hip fracture programs (SHFPs).^25^ Structured interdisciplinary fracture programs have in certain instances effectively decreased patient morbidity, shortened hospital stays, and enhanced functional recovery.^26,27,28,29^ However, there are concerns regarding the potential for bias in prescriptive practices to generate disparities in the implementation of high-quality, guideline-concordant care for patients after surgery for hip fracture. If present, disparities in receiving guideline-concordant care may contribute to the aforementioned racial and ethnic disparities in downstream surgical outcomes.

To our knowledge, there have been no studies within the literature to quantify differences in guideline-concordant care between racial and ethnic minority and nonminority older adults after surgery for hip fracture. There have also been no studies to quantify these differences while considering the intersectionality of race and ethnicity and gender. Therefore, this study aims to investigate (1) differences in the receipt of high-quality, guideline-concordant hip fracture surgery care between racial and ethnic minority patients and non-Hispanic White patients and (2) whether receiving care at an institution with an SHFP is associated with differential benefit in guideline-concordant care receipt for racial and ethnic minority and non-Hispanic White patients overall and when stratifying by gender. We hypothesize that there are disparities in guideline-concordant care between racial and ethnic minority and non-Hispanic White patients and that receiving care at an institution with an SHFP reduces disparities in care overall and when stratifying by gender.

## Methods

We queried the American College of Surgeons National Surgical Quality Improvement Program (ACS-NSQIP) Targeted Hip Fracture (THF) database from 2016 to 2021 for patients aged 65 years and older who received surgical fixation for hip fracture, excluding pathological fractures (either atypical or tumor-related). ACS-NSQIP case exclusion criteria included age younger than 18 years, combined trauma and transplant cases, return to the operating room that was related to an occurrence or complication of a prior procedure, and multiple NSQIP cases within 30 days. ACS-NSQIP excludes hospital sites with interrater reliability audit disagreement rate over 5% and may exclude hospitals that exhibit issues with either data quality or 30-day follow-up to ensure data integrity. The ACS-NSQIP database includes patient characteristics such as medical conditions at the time of surgery, demographics and many other variables collected from several participating hospitals. Over 700 hospitals participate in the ACS-NSQIP program, and a systematic sampling process was developed to limit selection bias in choosing cases for assessment.^30^ This cross-sectional study was exempt from institutional review board approval and did not require informed consent because the database is deidentified, in accordance with 45 CFR §46.

### Dependent Variables of Interest

The primary outcome of the study is receipt of guideline-concordant care. Specifically, this includes the likelihood of patients receiving postoperative bone-protection medication, being weight-bearing as tolerated on postoperative day 1 (WBAT-POD1), and being prescribed venous thromboembolism (VTE) prophylaxis medication.

WBAT-POD1 or earlier after hip fracture surgical fixation is a recommended intervention that improves balance, mobility, and discharge (to home) outcomes while reducing postoperative adverse events and complications that can result from extended immobility (eg, deep vein thrombosis [DVT]).^31,32^ ACS-NSQIP records WBAT-POD1 (yes, no, or not applicable) based on the presence or absence of clinical notes or orders documenting WBAT on the recovering limb occurred before the end of POD1. If WBAT-POD1 cannot be determined, the patient refuses to stand or walk, or there is an order for WBAT without evidence of WBAT on POD1 then NSQIP records the variable as “No.” The WBAT-POD1 variable is recorded as “N/A” for patients for whom it would be impossible (eg, based on ambulatory ability) or contraindicated due to other medical issues.

VTE prophylaxis is strongly recommended because of the high risk of DVT or pulmonary embolism (PE) in older adults after hip fracture.^31^ This may include the use of prophylactic medications or mechanical devices. Mechanical medical devices that provide static (eg, thrombo-embolic deterrent [TED] hose) or intermittent (eg, pneumatic sequential compression) or provide clot filtering (eg, inferior vena cava [IVC] filters) may also be used as VTE prophylaxis. The ACS-NSQIP captures new VTE prophylaxis prescriptions and continued use up to 28 days postoperatively (yes or no) for medical devices (excluding compression hose alone) or the following medications: aspirin (325 mg twice daily) alone or combined with another anticoagulant, heparin (unfractionated or low molecular weight), warfarin, fondaparinux sodium, oral factor 10A inhibitors, and direct thrombin inhibitor. ACS-NSQIP does not consider the use of the following medications alone (without the use of the previously mentioned medications) as VTE prophylaxis: clopidogrel, ticagrelor, ticlopidine, cilostazol, abciximab, eptifibatide, tirofiban, and dipyridamole. If a patient is receiving at least 2 medications out of any in the aforementioned list (eg, aspirin and clopidogrel), one of the medications on the list ACS-NSQIP allows to count alone toward prophylaxis, or uses a qualifying medical device during the VTE prophylaxis assessment window, they are categorized as having received VTE prophylaxis. Patients using prophylactic measures before surgery are also assessed for continued use postoperatively for this 28-day period. ACS-NSQIP records the postoperative VTE prophylaxis variable as yes for patients with evidence of prophylactic use for the entire 28-day period. If a patient is prescribed prophylactic measures postoperatively but is unable to complete the therapy through the entire 28-day period because of nonadherence, incident contraindication (eg, gastrointestinal bleed), or death, then ACS-NSQIP still records this variable as yes. If there is no clear evidence of prophylaxis, prophylaxis therapy is unknown, or the patient uses compression hose alone as prophylaxis, then ACS-NSQIP records the variable as no.

Initiating osteoporosis management as part of a postoperative interdisciplinary care program is strongly recommended in older adults after hip fracture.^31^ This includes the prescription of bone-protective medication, which is recommended to treat issues with bone fragility resulting from osteoporosis that may have contributed to the patient’s hip fracture. ACS-NSQIP records bone-protective medication as being prescribed postoperatively if a prescription for one of the following medications is given to the patient at any point within the 30 days after surgery: alendronate, risedronate, ibandronate, zoledronate, pamidronate, teriparatide, denosumab, raloxifene, vitamin D (D2 or D3 with at least 1000 IU per day), or calcitonin. Patients only taking herbal remedies, calcium preparations, calcium/vitamin D preparations with less than 1000 IU per day of vitamin D, or nonprescription multivitamins are recorded as not having been prescribed bone-protective medication.

### Independent Variables of Interest

Institutions participating in ACS-NSQIP report patient race and gender based on what is in the patient’s medical record. Race in the medical record may be patient self-assigned or assigned by institutional staff depending on the reporting institution’s internal practices.^33^ We grouped patient cases into 2 groups based on racial and ethnic social groups who have been historically sociopolitical advantaged (non-Hispanic White) and disadvantaged (racial and ethnic minority groups: American Indian or Alaska Native, Asian, Black or African American, Native Hawaiian or Pacific Islander, or multiple races and Hispanic ethnicity).^34^ For the years 2016 to 2020, ACS-NSQIP used a binary gender variable that only allowed institutions to report male or female. A nonbinary gender response option was added by ACS-NSQIP starting in 2021.

SHFPs implement processes to streamline perioperative hip fracture care via evidence-based institution-wide policies to improve outcomes and reduce adverse events.^25^ The ACS-NSQIP captures the use of SHFP at the hospital for the reported case (yes or no). Hospitals participating in ACS-NSQIP report whether they use an SHFP that includes standard hip fracture protocols. ACS-NSQIP records this variable as yes when an SHFP is in place at the institutional level, even if there is no evidence to whether the patient followed the standard protocol. This variable is recorded as no for hospitals that do not have an SHFP implemented, even if they use standard order sets.

### Statistical Analysis

All statistical analyses were conducted using SAS 9.4 (SAS Institute) from November 2022 to June 2024. After collecting demographic data, the missingness of race and ethnicity in the ACS-NSQIP THF data was relatively high, with notably higher missingness among patients receiving care at a hospital with an SHFP. Given the potential impact of high differential missingness of important study variables, we imputed missing race and ethnicity to inform racial and ethnic minority vs non-Hispanic White categorization using multiple imputation to reduce potential bias.^35^ Multiple imputation was implemented for missing categorical study variables using fully conditional specification (40 imputations). All independent and dependent variables were included in our imputation model; additionally, the size of the ACS-NSQIP cohort in our study also allowed us to include a large range of auxiliary variables in the imputation model that encompassed a wide range of pre- and postoperative information contained in the dataset, including: fracture type and pathological fracture status, operative information (eg, case type [elective, urgent, emergent], principal anesthesia technique American Society of Anesthesiologist classification), pre- and postoperative care variables (eg, origin of transfer, delirium status, use of mobility aids, adverse outcomes), and preoperative health information (eg, renal, cardiac, and cardiometabolic comorbidity history, body mass index, smoking status, functional status prior to surgery).

Analyses for our study were performed on the pooled multiply-imputed data. We estimated the probabilities of guideline-concordant care indicators by the binary race and ethnicity variable, calculating the risk difference (RD) between racial and ethnic minority and non-Hispanic White overall and stratified by care institution SHFP status. We further stratified these estimates by gender. We used modified Poisson regression to estimate measures of effect modification and their associated 95% CIs. We measured effect modification on the multiplicative (risk ratio) scale by estimating the ratio of risk ratios via an interaction term in the regression model and on the additive (risk difference) scale by estimating the interaction contrast.^36,37^ For the interaction contrast (IC) in this study, values greater than 0 indicate better outcomes of receiving care at an institution with a standardized hip fracture protocol in place among non-Hispanic White patients, and values less than 0 indicate better outcomes of receiving care at an institution with a standardized hip fracture protocol in place among racial and ethnic minority patients. The 95% CIs for the IC were calculated using the delta method.^38^ The statistical significance threshold was set at *P* < .05.

## Results

A total of 62 194 patients aged 65 years or older with hip fractures received surgical fixation within the ACS-NSQIP THF database from 2016 to 2021; 43 356 (69.7) patients were female; mean (SD) age was 82.4 (7.3) years. Preimputation demographic and outcome characteristics are presented in Table 1 overall and stratified by whether an SHFP was implemented at the institution where they received surgery. After multiple imputation, the estimated race distribution in our analytical sample was 0.6% (95% CI, 0.5%-0.7%) American Indian or Alaska Native, 3.2% (95% CI, 3.0%-3.5%) Asian, 0.1% (95% CI, 0.1%-0.1%) Native Hawaiian or Pacific Islander, 3.9% (95% CI, 3.7%-4.1%) Black or African American, 92.0% (95% CI, 91.7%-92.2%) White, 0.1% (95% CI, 0.1%-0.2% multiple races, and 0.1% (95% CI, 0.1%-0.1%) some other race; 3.3% (95% CI, 3.1%-3.4%) of patients were Hispanic. The analyzed sample after multiple imputation consisted of 11.2% (95% CI, 10.8%-11.5%) racial and ethnic minority patients, and the probability of receiving care at an SHFP was similar (risk difference, 0.2% [95% CI, −1.3%-1.6%]; *P* = .80) between racial and ethnic minority patients (64.2% [95% CI, 62.9%-65.6%]) and non-Hispanic White patients (64.4% [95% CI, 64.0%-64.8%]).

**Table 1.** Characteristics of Older Adults Who Received Hip Fracture Surgical Fixation by Institutional Standardized Hip Fracture Care Program Status, ACS-NSQIP Targeted Hip Fracture Data, 2016-2021

Characteristic	Original data				Multiply imputed data			
	Standardized hip fracture care program		Total (n = 62194)	*P* value	Standardized hip fracture care program		Total (n = 62194)	*P* value
	Yes (n = 40048)	No (n = 22146)			Yes (n = 40048)	No (n = 22146)		
Age, y								
Mean (SD)	82.6 (7.25)	82.1 (7.41)	82.4 (7.31)	<.001^a^	NA	NA	NA	NA
Median (range)	85.0 (65.0-90.0)	84.0 (65.0-90.0)	84.0 (65.0-90.0)		NA	NA	NA	NA
Gender, No. (%)								
Female	27 802 (69.4)	15 554 (70.2)	43 356 (69.7)	.10^b^	NA	NA	NA	NA
Male	12 245 (30.6)	6591 (29.8)	18 836 (30.3)		NA	NA	NA	NA
Nonbinary	1 (0.0)	1 (0.0)	2 (0.0)		NA	NA	NA	NA
Race, No. (%)								
American Indian or Alaska Native	46 (0.1)	177 (0.8)	223 (0.4)	<.001^b^	166 (0.4)	206 (0.9)	372 (0.6)	<.001^b^^,^^c^
Asian	424 (1.1)	478 (2.2)	902 (1.5)		1403 (3.5)	610 (2.8)	2014 (3.2)	
Black or African American	868 (2.2)	687 (3.1)	1555 (2.5)		1623 (4.1)	788 (3.6)	2410 (3.9)	
Native Hawaiian or Pacific Islander	19 (0.0)	14 (0.1)	33 (0.1)		44 (0.1)	17 (0.1)	61 (0.1)	
White	20847 (52.1)	18297 (82.6)	39144 (62.9)		36715 (91.7)	20485 (92.5)	57200 (92.0)	
Some other race	33 (0.1)	29 (0.1)	62 (0.1)		33 (0.1)	29 (0.1)	62 (0.1)	
Multiple races	6 (0.0)	3 (0.0)	9 (0.0)		64 (0.2)	11 (0.0)	75 (0.1)	
Missing	17 805 (44.5)	2461 (11.1)	20 266 (32.6)		0	0	0	
Hispanic ethnicity, No. (%)	1042 (2.6)	808 (3.6)	1850 (3.0)	<.001^b^	1168 (2.9)	861 (3.9)	2029 (3.3)	<.001^b^^,^^c^
Missing	17 677 (44.1)	2633 (11.9)	20 310 (32.7)		0	0	0	
Racial and ethnic minority, No. (%)	2393 (6.0)	2156 (9.7)	4549 (7.3)	<.001^b^	4456 (11.1)	2482 (11.2)	6938 (11.2)	0.60^b^^,^^c^
Missing	18 108 (45.2)	2881 (13.0)	20 989 (33.7)		0	0	0	
Type/location of fracture, No. (%)								
Femoral neck fracture (subcapital Garden types 1 and 2) undisplaced	3657 (9.1)	1734 (7.8)	5391 (8.7)	<.001^b^	NA	NA	NA	NA
Femoral neck fracture (subcapital Garden types 3 and 4) displaced	12 292 (30.7)	6795 (30.7)	19 087 (30.7)		NA	NA	NA	
Intertrochanteric	21 434 (53.5)	12 020 (54.3)	33 454 (53.8)		NA	NA	NA	
Subtrochanteric	2133 (5.3)	1127 (5.1)	3260 (5.2)		NA	NA	NA	
Other/cannot be determined	532 (1.3)	470 (2.1)	1002 (1.6)		NA	NA	NA	
WBAT-POD1, No. (%)								
Yes	27 416 (68.5)	14 395 (65.0)	41 811 (67.2)	<.001^b^	27534 (68.8)	14508 (65.5)	42043 (67.6)	<.001^b^^,^^c^
No	8146 (20.3)	5206 (23.5)	13 352 (21.5)		8179 (20.4)	5240 (23.7)	13419 (21.6)	
NA (bed-ridden or other medical issues)	4321 (10.8)	2382 (10.8)	6703 (10.8)		4335 (10.8)	2397 (10.8)	6732 (10.8)	
Missing	165 (0.4)	163 (0.7)	328 (0.5)		0	0	0	
Prescription of postoperative bone-protection medication, No. (%)	24 187 (60.4)	9434 (42.6)	33 621 (54.1)	<.001^b^	NA	NA	NA	NA
Medical VTE prophylaxis continued 28 d postoperative, No. (%)	27 012 (67.4)	13 161 (59.4)	40 173 (64.6)	<.001^b^	27121 (67.7)	13262 (59.9)	40383 (64.9)	<.001^b^^,^^c^
Missing	166 (0.4)	164 (0.7)	330 (0.5)		0	0	0	

Abbreviations: ACS-NSQIP, American College of Surgeons National Surgical Quality Improvement Program; NA, not applicable; VTE, venous thromboembolism; WBAT-POD1, weight-bearing as tolerated on postoperative day 1.

^a^ Two-sample *t* test.

^b^ χ^2^ test.

^c^ Pooled χ^2^ test across multiply imputed data.

When comparing racial and ethnic minority and non-Hispanic White patients, racial and ethnic minority patients had a 2.1% (95% CI, 0.6%-3.7%; *P* = .006) lower probability of receiving postoperative bone-protective medication (52.2% [95% CI, 50.8%-53.6%]) than non-Hispanic White patients (54.3% [95% CI, 53.9%-54.7%]) and a 2.5% (95% CI, 1.2%-3.9%; *P* < .001) lower probability of being WBAT-POD1 (73.6% [95% CI, 72.3%-74.8%) than non-Hispanic White patients (76.1% [95% CI, 75.7%-76.5%]). The probability of receiving VTE prophylaxis was similar (risk difference, 0.1% [95% CI, −1.3% to 1.5%]; *P* = .89) between racial and ethnic minority patients (64.8% [95% CI, 63.5%-66.2%]) and non-Hispanic White patients (64.9% [95% CI, 63.5%-66.2%]).

Both racial and ethnic minority and non-Hispanic White patients had a higher probability of receiving guideline-concordant care if their institution had an SHFP. Racial and ethnic minority patients were 14.9% (95% CI, 12.1%-17.8%; *P* < .001) more likely to be prescribed bone-protective medication, 3.0% (95% CI, 0.4%-5.6%; *P* = .03) more likely to be WBAT-POD1, and 6.6% (95% CI, 4.0%-9.3%; *P* < .001) more likely to be appropriately prescribed VTE prophylaxis medication in an SHFP compared with no SHFP. Non-Hispanic White patients were 18.1% (95% CI, 17.3%-19.0%; *P* < .001) more likely to be prescribed bone-protective medication, 3.7% (95% CI, 2.9%-4.5%; *P* < .001) more likely to be WBAT-POD1, and 8.0% (95% CI, 7.1%-8.9%; *P* < .001) more likely to be appropriately prescribed VTE prophylaxis medication in an SHFP compared with no SHFP. Furthermore, differences by race and ethnicity were observed within institutions implementing SHFPs. Non-Hispanic White patients in SHFPs had a 2.8% (95% CI, 1.0%-4.6%; *P* = .003) higher probability of achieving WBAT-POD1 and a 3.2% (95% CI, 1.2%-5.3%) higher probability of receiving bone-protective medication compared with racial and ethnic minority patients (Table 2).

**Table 2.** Association Between Racial and Ethnic Minority Status and Postoperative Guideline-Concordant Care by Institutional Standardized Hip Fracture Protocol Status

Guideline-concordant care outcome	No SHFP				SHFP				Risk difference for SHFP within race and ethnicity category^a^			
	Probability of receipt^a^		Risk difference within SHFP strata^a^	*P* value	Probability of receipt^a^		Risk difference within SHFP strata^a^	*P* value				
	Racial and ethnic minority	Non-Hispanic White			Racial and ethnic minority	Non-Hispanic White			Racial and ethnic minority	*P* value	Non-Hispanic White	*P* value
Postoperative bone-protective medication	0.426 (0.405 to 0.447)	0.426 (0.419 to 0.433)	0.000 (−0.022 to 0.022)	.98	0.575 (0.556 to 0.593)	0.608 (0.602 to 0.613)	0.032 (0.012 to 0.053)	.002	0.149 (0.121 to 0.178)	.001	0.181 (0.173 to 0.190)	.001
WBAT-POD1	0.716 (0.697 to 0.736)	0.737 (0.730 to 0.744)	0.021 (0.000 to 0.042)	.06	0.746 (0.730 to 0.763)	0.774 (0.769 to 0.779)	0.028 (0.010 to 0.046)	.003	0.030 (0.004 to 0.056)	.03	0.037 (0.029 to 0.045)	.001
VTE prophylaxis	0.606 (0.585 to 0.626)	0.598 (0.591 to 0.605)	−0.007 (−0.030 to 0.014)	.50	0.672 (0.655 to 0.683)	0.678 (0.673 to 0.683)	0.005 (−0.013 to 0.024	.54	0.066 (0.040 to 0.093)	.001	0.080 (0.071 to 0.089)	.001

Abbreviations: SHFP, Standardized Hip Fracture Protocol; VTE, venous thromboembolism; WBAT-POD1, weight-bearing as tolerated on postoperative day 1.

^a^ Point estimate (95% CI).

When analyzing the association of racial and ethnic minority status with receiving guideline-concordant care, we found that SHFPs were associated with greater benefit for non-Hispanic White patients in being prescribed postoperative bone-protection medication (IC, 0.033 [95% CI, 0.005-0.060]; *P* = .02). However, there were no significant benefits associated between racial and ethnic minority and non-Hispanic White and postoperative guideline-concordant care by institutional SHFP status in achieving WBAT-POD1 and receiving VTE prophylaxis (Table 3).

**Table 3.** Association Between Racial and Ethnic Minority Status and Postoperative Guideline-Concordant Care by Institutional Standardized Hip Fracture Protocol Status

Guideline-concordant care outcome	Interaction contrast^a^^,^^b^	*P* value	Ratio of risk ratios^a^^,^^c^	*P* value
Postoperative bone-protective medication	0.033 (0.005 to 0.060)	.02	1.06 (0.99 to 1.13)	.09
WBAT-POD1	0.007 (−0.019 to 0.034)	.59	1.01 (0.97 to 1.05)	.67
VTE prophylaxis	0.014 (−0.013 to 0.040)	.33	0.98 (0.94 to 1.02)	.35

Abbreviations: VTE, venous thromboembolism; WBAT-POD1, weight-bearing as tolerated on postoperative day 1.

^a^ Point estimate (95% CI).

^b^ Measure of estimated interaction effect on the additive (risk difference) scale; values greater than 0 indicate beneficial outcome of receiving care at an institution with a standardized hip fracture protocol in place is higher among non-Hispanic White patients; values less than 0 indicate beneficial outcome of receiving care at an institution with a standardized hip fracture protocol in place is higher among racial or ethnic minority patients.

^c^ Measure of estimated interaction effect on the multiplicative (relative risk) scale; reference category was racial or ethnic minority patients receiving care at institutions without standardized hip fracture protocols.

Receiving care at an institution with an SHFP was associated with even larger improvements in providing guideline-concordant care when examining differences within gender categories. For racial and ethnic minority female patients not in SHFPs, the probability of being WBAT-POD1 was lower on an absolute scale for racial and ethnic minority female patients than non-Hispanic White female patients (risk difference, 2.7% [95% CI, 0.1%-5.2%]; *P* = .04), but SHFP was associated with a decrease in this difference (risk difference, 2.3% [95% CI, 0.1%-4.4%]; *P* = .04). Non-Hispanic White female patients had a higher probability of postoperative bone-protective medication (risk difference, 3.1% [95% CI, 0.7%-5.6%]; *P* = .01) and WBAT-POD1 (risk difference, 2.3% [95% CI, 0.1%-4.4%], *P* = .04) compared with racial and ethnic minority within hospitals that have SHFP. When an SHFP was implemented, there was an increase in the probability of WBAT-POD1 among racial and ethnic minority female patients (risk difference, 4.1% [95% CI, 1.0%-7.1%], *P* = .009) compared with the increase in likelihood with non-Hispanic White female patients (risk difference, 3.7% [95% CI, 2.7%-4.6%], *P* < .001). There were no significant differences in the probability of VTE prophylaxis between racial and ethnic minority and non-Hispanic White females within an SHFP strata. However, when an SHFP was implemented, there was a higher probability of VTE prophylaxis among racial and ethnic minority female patients (risk difference, 6.9% [95% CI, 3.7%-10.2%], *P* < .001) compared with the increase in likelihood with non-Hispanic White female patients (risk difference, 8.4% [95% CI, 7.4%-9.4%], *P* < .001) (Table 4).

**Table 4.** Association Between Racial and Ethnic Minority Status and Postoperative Guideline-Concordant Care by Institutional Standardized Hip Fracture Protocol Status, Stratified by Gender

Guideline-concordant care outcome	No SHFP				SHFP					Risk difference for SHFP within race and ethnicity category^a^			
	Probability of receipt^a^		Risk difference within SHFP strata^a^	*P* value	Probability of receipt^a^			Risk difference within SHFP strata^a^	*P* value				
	Racial and ethnic minority	Non-Hispanic White			Racial and ethnic minority		Non-Hispanic White			Racial and ethnic minority	*P* value	Non-Hispanic White	*P* value
**Female**													
Postoperative bone-protective medication	0.457 (0.432 to 0.482)	0.453 (0.445 to 0.462)	−0.004 (−0.031 to 0.023)	.78	0.598 (0.575 to 0.621)	0.629 (0.623 to 0.636)		0.031 (0.056 to 0.007)	.01	0.141 (0.107 to 0.175)	.001	0.176 (0.166 to 0.187)	.001
WBAT-POD1	0.710 (0.687 to 0.734)	0.737 (0.729 to 0.745)	0.027 (0.001 to 0.052)	.04	0.751 (0.731 to 0.771)	0.774 (0.768 to 0.779)		0.023 (0.001 to 0.044)	.04	0.041 (0.010 to 0.071)	.009	0.037 (0.027 to 0.046)	.001
VTE prophylaxis	0.606 (0.581 to 0.631)	0.593 (0.585 to 0.602)	−0.012 (−0.039 to 0.014)	.36	0.675 (0.655 to 0.695)	0.677 (0.671 to 0.683)		0.002 (−0.02 to 0.024)	.86	0.069 (0.037 to 0.102)	.001	0.084 (0.074 to 0.094)	.001
**Male**													
Postoperative bone-protective Medication	0.355 (0.320 to 0.391)	0.362 (0.349 to 0.374)	0.006 (−0.032 to 0.044)	.75	0.528 (0.498 to 0.557)	0.557 (0.548 to 0.567)		0.030 (−0.002 to 0.062)	.07	0.172 (0.126 to 0.219)	.001	0.196 (0.18 to 0.211)	.001
WBAT-POD1	0.730 (0.694 to 0.766)	0.737 (0.725 to 0.749)	0.007 (−0.032 to 0.045)	.73	0.736 (0.707 to 0.766)	0.775 (0.766 to 0.784)		0.038 (0.007 to 0.070)	.02	0.006 (−0.040 to 0.053)	.79	0.038 (0.023 to 0.053)	.001
VTE prophylaxis	0.606 (0.569 to 0.642)	0.609 (0.596 to 0.621)	0.003 (−0.036 to 0.042)	.88	0.666 (0.636 to 0.696)	0.679 (0.67 to 0.688)		0.013 (−0.019 to 0.046)	.41	0.06 (0.014 to 0.107)	.11	0.071 (0.055 to 0.086)	.001

Abbreviations: SHFP, Standardized Hip Fracture Protocol; VTE, venous thromboembolism; WBAT-POD1, weight-bearing as tolerated on postoperative day 1.

^a^ Point estimate (95% CI).

Among male patients not in SHFPs, there were no significant differences between racial and ethnic minority and non-Hispanic White among being prescribed postoperative bone-protective medication, WBAT-POD1, or VTE prophylaxis. However, in male patients within hospitals that have SHFPs, there were differences in being WBAT-POD1 (risk difference, 3.8% [95% CI, 0.7%-7.0%]; *P* = .02), indicating a higher probability among non-Hispanic White patients. There were no significant differences in being prescribed postoperative bone-protective medication or VTE prophylaxis for male patients in a hospital with an SHFP between racial and ethnic minority and non-Hispanic White patients. Across SHFP strata, non-Hispanic White patients had more improvement than racial and ethnic minority patients in being prescribed postoperative bone-protective medication (risk difference, 19.6% [95% CI, 18.0%-21.1%]; *P* < .001), WBAT-POD1 (risk difference, 3.8% [95% CI, 2.3%-5.3%]; *P* < .001), and VTE prophylaxis (risk difference, 7.1% [95% CI, 5.5%-8.6%]; *P* < .001) (Table 4).

When examining the association of racial and ethnic minority status with guideline-concordant care for female patients, we found that SHFPs were associated with better outcomes for non-Hispanic White females in terms of being prescribed postoperative bone-protection medication (IC, 0.035 [95% CI, 0.002-0.068]; *P* = .04). However, for females there were no significant benefit observed between racial and ethnic minority and non-Hispanic White and postoperative guideline-concordant care by institutional SHFP status in achieving WBAT-POD1 and receiving VTE prophylaxis. For males, SHFPs also showed there were no significant benefits for non-Hispanic White patients in being prescribed postoperative bone-protection medication and VTE prophylaxis, while racial and ethnic minority males benefited more from WBAT-POD1 under SHFPs (Table 5).

**Table 5.** Interaction Contrast Between Racial and Ethnic Minority Status and Postoperative Guideline-Concordant Care by Institutional Standardized Hip Fracture Protocol Status, Stratified by Gender

Guideline-concordant care outcome	Interaction contrast^a^^,^^b^	*P* value	Ratio of risk ratios^a^^,^^c^	*P* value
**Female**				
Postoperative bone-protective medication	0.035 (0.002 to 0.068)	.04	1.06 (0.99 to 1.14)	.10
WBAT-POD1	−0.004 (−0.036 to 0.028)	.04	0.99 (0.95 to 1.04)	.76
VTE prophylaxis	0.014 (−0.018 to 0.046)	.38	1.02 (0.97 to 1.08)	.40
**Male**				
Postoperative bone-protective medication	0.023 (−0.023 to 0.070)	.32	1.04 (0.92 to 1.17)	.55
WBAT-POD1	0.032 (−0.015 to 0.078)	.18	1.04 (0.97 to 1.12)	.23
VTE prophylaxis	0.014 (−0.038 to 0.059)	.67	1.02 (0.94 to 1.10)	.71

Abbreviations: WBAT-POD1, weight-bearing as tolerated on postoperative day 1; VTE, venous thromboembolism.

^a^ Point estimate (95% CI).

^b^ Measure of estimated interaction effect on the additive (risk difference) scale; values greater than 0 indicate beneficial outcome of receiving care at an institution with a standardized hip fracture protocol in place is higher among non-Hispanic White patients; values less than 0 indicate beneficial outcome of receiving care at an institution with a standardized hip fracture protocol in place is higher among racial or ethnic minority patients.

^c^ Measure of estimated interaction effect on the multiplicative (relative risk) scale; reference category is racial or ethnic minority patients receiving care at institutions without standardized hip fracture protocols.

## Discussion

Guideline-concordant care, such as medical comanagement, SHFPs, and care coordination, has effectively improved patient outcomes following hip fracture surgery.^26,27,28,29^ Our study underscores the existence of disparities in the implementation of hospital-based measures for racial and ethnic minority patients who have undergone hip fracture surgery. Non-Hispanic White patients were more likely to receive guideline-concordant care for postoperative bone-protective medication and WBAT-POD1 compared with racial and ethnic minority patients, with overall low proportions of patients receiving these interventions. Participation in SHFPs was associated with greater likelihood of receiving guideline-concordant care across all patients, but it was also associated with greater disparities between non-Hispanic White and racial and ethnic minority patients. While SHFPs have the strongest association with greater bone-protective medication receipt, they have a similar association with greater VTE prophylaxis receipt across race and ethnicity groups. Gender-stratified results show similar patterns, with a notably low proportion of male patients receiving postoperative bone-protective medication, especially in non-SHFP institutions. Although previous studies have investigated racial and ethnic disparities within orthopedic trauma, the gap in the prescriptive practices of guideline-concordant care for patients undergoing hip fracture surgery has, to our knowledge, not been reported until now.

SHFPs have been used to optimize care for patients with hip fractures, particularly among older adult populations.^27,39^ These programs typically consist of components such as admission checklists, perioperative orders, multidisciplinary evaluation and management, rehabilitation goals, and specific discharge criteria. Implementing SHFPs has been demonstrated to reduce both the costs ($18 706 to $13 755; *P* < .001) and complications associated with hip fractures.^40^ In addition, Kates et al found that utilizing an organized hip fracture program resulted in substantial savings across various expenditure areas, with costs 66.7% lower than the expected national costs alongside lower-than-national averages within length of stay, mortality, complications, and readmissions. There the racial disparities identified in this study have implications for hip fracture care standards.^27^

There is a 2.2% incidence of symptomatic DVT following hip fracture surgery, with 85% occurring within 5 weeks postoperatively.^41^ Black patients were found to have a higher risk of DVT, VTE, and pulmonary embolism compared with other racial and ethnic groups within orthopedic surgery.^42,43,44,45,46^ The implementation of other standard-of-care perioperative interventions, such as SHFPs that include DVT prophylaxis order sets, early ambulation, and intensive physical therapy, may help reduce the risk of postoperative DVT.^47,48,49^ Enrollment in SHFPs might also contribute to improved adherence to DVT prophylaxis medication. This is consistent with our findings that racial and ethnic minority patients were less likely to be prescribed appropriate DVT prophylaxis; however, a reduction in that disparity was associated with the implementation of an SHFP.

Approximately 10% of older adults with hip fractures experience a subsequent hip fracture within 1 to 5 years after their initial fall.^50,51^ The majority of patients who experience a hip fracture do not use osteoporosis medication in the following year.^52^ Despite data recommending that orthopaedic surgeons play an active role in the management of osteoporosis to improve the rate of treatment at 6 months following a hip fracture,^53^ Kristensen et al found that less than one-third of hip fracture patients were prescribed antiosteoporosis medication within a year after fracture regardless of patient demographics.^54^ In addition, Black and Hispanic patients have a higher incidence of hip fractures among individuals under 75 years of age compared with White patients with hip fractures older than 75 years in the southwestern United States.^20^ This historical disparity highlights our findings that racial and ethnic minority patients were less likely to receive prescriptions for bone-protective medications. While SHFP institutions generally provided higher quality care for both racial and ethnic minority and non-Hispanic White patients, non-Hispanic White patients seemed to receive a disproportionate benefit in some of our outcomes of interest, indicating a net benefit but also a continued disparity. It is important to note that the current clinical practice guidelines do not strongly support the routine use of antiresorptive medications for every patient. While discussions, further workups, laboratory tests, and shared decision-making regarding these medications may be beneficial, there is insufficient evidence to recommend their use universally. This lack of robust evidence for antiresorptive medications may impact how institutions incorporate them into hip fracture protocols, potentially influencing disparities in care. Consequently, further research is essential to establish clearer evidence for their use and to guide future clinical practice.

Early weight-bearing can reduce the incidence of postoperative complications, such as fragility fractures and delirium,^55,56^ while early mobilization can decrease mortality among patients with hip fracture surgical fixation by 10.5%.^57^ In addition, enabling prompt mobilization and unrestricted weight bearing in older patients following hip fracture surgery reduced hospitalization duration.^58^ In our study, we noted that racial and ethnic minority patients were less likely to achieve WBAT-POD1. This disparity not only persisted but was slightly larger at institutions with an SHFP.

Overall, the probability of achieving better guideline-concordant care outcomes, and thereby implying better quality care, was much higher in institutions with SHFPs. Both racial and ethnic minority and non-Hispanic White patients had higher probability of receiving essential postoperative interventions in SHFP facilities, such as bone-protective medication and VTE prophylaxis. However, disparities persisted, with non-Hispanic White patients consistently receiving higher rates of guideline-concordant care. Receiving care at an institution with SHFPs was associated with significantly higher chances of receiving bone-protective medication and WBAT-POD1 for all patients, but the gap between racial and ethnic minority and non-Hispanic White patients slightly widened. Gender-stratified analyses showed improvements for both males and females, yet non-Hispanic White patients still benefited more. These findings highlight the crucial role of SHFPs in enhancing care quality and adherence to clinical guidelines while emphasizing the need to address disparities to ensure equitable health care delivery.

### Limitations

Our study has limitations. The ACS-NSQIP THF database only includes 30-day outcomes, which prevents us from analyzing long-term postoperative outcomes related to the disparity in prescribed quality measures. There is a high rate of missingness in the original ACS-NSQIP data for race and ethnicity that was at least crudely related to whether care was received at an institution with an SHFP. This differential missingness increases the chance for bias in results if using complete case analysis. To address this issue, we handled missing values for all analyses via multiple imputation. The database does not provide information on region, payor status, or other social determinants of health, limiting our ability to understand the influence of these factors on the disparity of quality measures. In addition, our analyses did not attempt to adjust for other covariates, as any other covariate in the ACS-NSQIP database would likely be an intermediate or mediator of the associations explored in this study rather than a confounder^59^; fully exploring the reasons for observed differential receipt of guideline-concordant care was beyond the scope of this study. The ACS-NSQIP THF database also lacks detailed descriptions of the standardized protocols and does not provide the ability to aggregate information at the institution level. Therefore, it is unclear the variability that could potentially exist between the participating institutions. Another limitation of this database pertains to its generalizability, as participating institutions, potentially limiting the extrapolation of findings to other types of health care facilities not represented within the program. There may also be potential issues with accuracy and data recording in the NSQIP dataset; however, ACS-NSQIP takes steps to limit errors in data recording and we assume any residual error would be nondifferential with respect to our study question, meaning the impact of any residual bias from recording error in our results would be toward the null.

## Conclusions

Older adults who received care at an institution with an SHFP were more likely to receive guideline-concordant care (bone-protective medication, WBAT-POD1, and VTE prophylaxis), regardless of race and ethnicity group. However, the probability of receiving guideline-concordant care at an institution with an SHFP was generally higher for non-Hispanic White patients than racial and ethnic minority patients.

## Notes

Supplement.
Data Sharing Statement
