# Supplementary material for: Racial and Ethnic Disparities in Providing Guideline-Concordant Care After Hip Fracture Surgery
Source: JAMA Netw Open. 2024 Aug 27;7(8):e2429691. doi: 10.1001/jamanetworkopen.2024.29691 (PMC11350472; doi:10.1001/jamanetworkopen.2024.29691)
Supplement: Supplement. — Data Sharing Statement [file jamanetwopen-e2429691-s001.pdf]

# Data Sharing Statement

Bethell. Racial and Ethnic Disparities in Providing Guideline-Concordant Care After Hip Fracture Surgery. *JAMA Netw Open*. Published August 22, 2024.
doi:10.1001/jamanetworkopen.2024.29691

**Data**
**Data available:** No

**Additional Information**
**Explanation for why data not available:** The data utilized for this study is from the ACS-NSQIP database which requires permission from the ACS for utilization and sharing.
